# Association between cumulative atherogenic index of plasma and dementia risk score in middle-aged and elderly adults: a longitudinal analysis of the CHARLS cohort

**DOI:** 10.1186/s40001-025-03638-5

**Published:** 2025-12-07

**Authors:** Tongjie Zhang, Qian Xu, Jie Zhou

**Affiliations:** 1 Master of Medicine, resident physician, ICU, Wujiang Traditional Chinese Medicine Hospital, Suzhou, 215200 Jiangsu China; 2 Department of Encephalopathy, Wujiang Traditional Chinese Medicine Hospital, Suzhou, 215200 Jiangsu China

**Keywords:** Cumulative atherogenic index of plasma, Dementia risk score, Linear regression model, Visualization

## Abstract

**Background:**

Dementia has become an important public health challenge as the aging population in China accelerates, highlighting the need to identify modifiable risk factors. The cumulative atherogenic index of plasma (CumAIP), a marker reflecting long-term dyslipidemia, may contribute to dementia via vascular and inflammatory pathways, but longitudinal evidence in Chinese adults around 60 years of age remains scarce.

**Methods:**

Data were extracted from three waves of the China Health and Retirement Longitudinal Study (CHARLS): 2012 (Wave 1), 2015 (Wave 3), and 2018 (Wave 4). A total of 6473 participants with complete AIP measurements at 2012 and 2015 were included; CumAIP was computed as the time-weighted average of AIP values, normalized by the 2012–2015 observation duration. Dementia risk (primary outcome) was assessed via the Rotterdam Basic Dementia Risk Model (BDRM) using 2018 data, reflecting a 3-year follow-up with 2015 as the baseline. To validate the BDRM, we conducted a secondary analysis: Spearman rank correlation between 2018 BDRM scores and 2015 CHARLS cognitive function scores. Statistical analyses included: multivariable linear regression with three progressive adjustment tiers (sociodemographic, clinical, lifestyle factors); restricted cubic spline (RCS) curves (3 knots at 10th/50th/90th CumAIP percentiles) for linearity testing; quartile-based analyses (CumAIPQ1–Q4, Q1 as reference) for dose–response relationships; and subgroup (by gender, age, residence, and lifestyle/metabolic factors) and sensitivity analyses [multiple imputation, complete-case analysis, linear regression of CumAIP vs. 2015 cognitive function scores, linear regression of low-density lipoprotein cholesterol (LDL-C) vs. BDRM scores] to validate robustness.

**Results:**

First, the 2018 BDRM score was significantly negatively correlated with the 2015 cognitive function score (Spearman’s *r* = − 0.26, *P* < 0.001). Higher CumAIP was associated with elevated BDRM scores, remaining significant after full adjustment (*β* = 0.058, 95% CI 0.018–0.098, *P* = 0.004). RCS confirmed a linear relationship (nonlinear term *P* = 0.9671), and quartile analyses showed a dose–response trend (Q4 vs. Q1, *β* = 0.103, 95% CI 0.009–0.197, *P* = 0.031). Subgroup effects were more pronounced in females, those aged ≥ 60 years, rural residents, nondrinkers, smokers, and BMI ≥ 24 kg/m^2^. Sensitivity analyses validated robustness: (1) CumAIP correlated positively with 2015 cognitive function (*β* = 0.123, 95% CI 0.008–0.237, *P* = 0.037); (2) LDL-C correlated positively with BDRM scores (*β* = 0.019, 95% CI 0.009–0.028, *P* = 0.0001), with a smaller effect than CumAIP.

**Conclusion:**

Cumulative elevated CumAIP is independently associated with higher BDRM-estimated dementia risk in Chinese adults (mean age 61.4 ± 8.5 years), with linear and dose-dependent relationships. The association is more prominent in high-risk subgroups: females, aged ≥ 60 years, rural residents, nondrinkers, smokers, and BMI ≥ 24 kg/m^2^. The BDRM’s validity supports its reliability. CumAIP’s stronger association with dementia risk than LDL-C underscores its value as a comprehensive lipid marker for risk stratification. Dynamic lipid monitoring to maintain low CumAIP may aid dementia prevention in these high-risk groups.

## Background

Dementia, a progressive neurodegenerative syndrome characterized by cognitive decline and functional impairment, represents a critical public health challenge with global prevalence exceeding 55 million cases [1]. In China, accelerated population aging is projected to expand the elderly cohort to 483 million by 2050 [2], potentially elevating dementia prevalence to 21.77% in this demographic [3]. The resultant socioeconomic burden exceeds 1% of annual GDP, underscoring the urgency for effective preventive strategies [3].

Although established risk factors include advanced age, genetic predisposition, vascular pathologies, and metabolic disorders [1, 4], approximately 30–40% of dementia cases lack these traditional determinants [5]. This knowledge gap necessitates the identification of more specific, dynamic biomarkers for precision prevention—especially markers that capture the long-term cumulative effects of metabolic dysfunction, which are often missed by single-time-point measurements of traditional risk factors.

Atherosclerosis increases the risk of cardio-cerebrovascular diseases, and dyslipidemia (a core, modifiable component of metabolic disorders) is a key driver [6]. However, individual lipid markers (e.g., total cholesterol (TC), low-density lipoprotein cholesterol (LDL-C), high-density lipoprotein cholesterol (HDL-C)) have limitations: they reflect only a single aspect of lipid metabolism and fail to quantify the overall atherogenic potential of plasma. This is where the AIP offers unique value: as the logarithmic transformation of the TG/HDL-C ratio, AIP integrates pro-atherogenic (TG-rich) and anti-atherogenic (HDL-C-rich) lipoproteins to provide a comprehensive measure of dyslipidemic burden [7]. First proposed by Dobiasova and Frohlich, AIP has been validated to correlate more strongly with coronary atherosclerosis (*r* = 0.62) than individual lipids like LDL-C (*r* = 0.38) or TC (*r* = 0.29), confirming its superiority as an exploratory index for atherogenic risk [8].

Notably, this atherogenic risk-assessing value of AIP is also relevant to cognitive outcomes: studies have shown that elevated AIP levels correlate with a higher risk of cognitive impairment in adults around 60 years of age [9], aligning with the age profile of our CHARLS-based study population. However, relying solely on baseline AIP values—without considering the cumulative effects of time-varying lipid profiles—may overlook the impact of long-term dyslipidemia. This is critical for dementia research, as neurodegenerative processes (e.g., *β*-amyloid deposition) develop over decades, and short-term lipid measurements cannot capture the chronic exposure that drives these changes. Prospective cohort study has shown that CumAIP—is closely related to the progression and outcome of prediabetes, and is independently associated with cardiometabolic disease (CMD) [10, 11]. Importantly, CumAIP addresses a key limitation of baseline AIP: by normalizing repeated AIP measurements to the total follow-up duration, it captures the chronicity of dyslipidemia—a feature that aligns with the decades-long pathophysiology of dementia.

Although atherogenic dyslipidemia is implicated in dementia pathogenesis, existing studies suffer from three critical limitations: (1) reliance on cross-sectional AIP and arterial stiffness measurements [12]; (2) lack of Asian population data;(3) the lack of direct validation by biomarkers (such as *β*-amyloid and Tau proteins) has led to insufficient exploration of the underlying mechanisms. The CHARLS cohort—with its repeated lipid profiling (2012–2015), 3-year follow-up for dementia risk assessment (2018), and nationally representative sampling of Chinese adults aged ≥ 45 years—provides a unique opportunity to address these gaps. Specifically, CHARLS’repeated AIP measurements allow us to calculate CumAIP (time-weighted cumulative AIP exposure), while its detailed demographic and clinical data enable rigorous adjustment for confounders (e.g., metabolic disorders, vascular pathologies) that could obscure the CumAIP–dementia association.

Therefore, this study used CHARLS data to: (1) Investigate the longitudinal association between CumAIP and dementia risk scores (assessed by the BDRM) in Chinese adults around 60 years of age; (2) Validate the linearity and dose–response relationship of this association; (3) Evaluate CumAIP’s incremental value for dementia risk stratification—beyond traditional risk factors like metabolic disorders and single-time-point lipids.

## Methods

### Study population and design

**Fig. 1:**
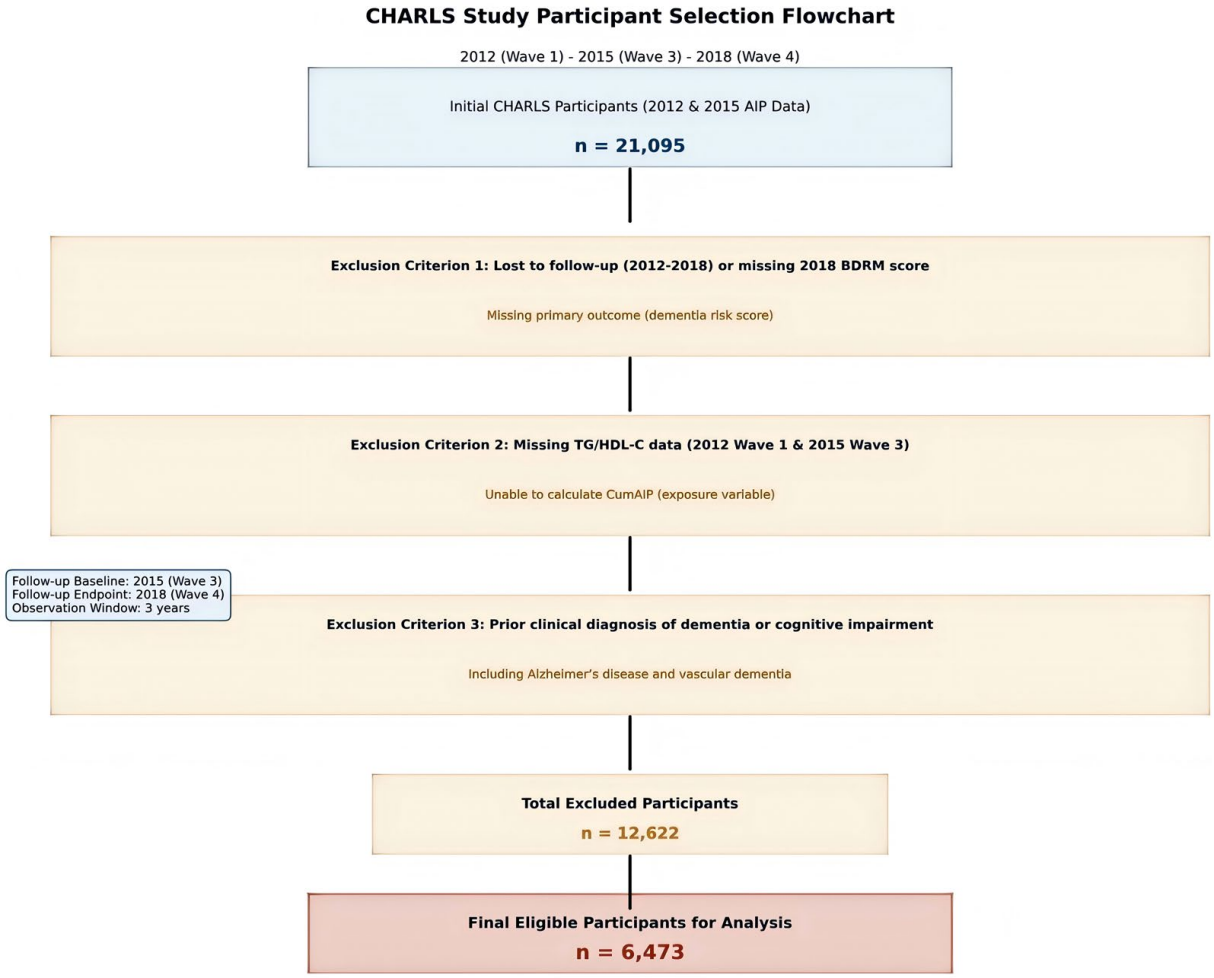
Flow chart of study participants. *BDRM* Basic Dementia Risk Model; *CHARLS* China Health and Retirement Longitudinal Study; *CumAIP* Cumulative Atherogenic Index of Plasma

CHARLS, initiated in 2011, is a prospective nationwide cohort with biennial follow-ups, targeting Chinese adults aged ≥ 45 years to track long-term health changes. For this study—aimed at exploring the longitudinal association between CumAIP and dementia risk—data from three CHARLS waves were extracted: 2012 (Wave 1) and 2015 (Wave 3) for CumAIP calculation (Sect. "Calculation of CumAIP"), and 2018 (Wave 4) for assessing the primary outcome (dementia risk score) [13, 14]. Data collection followed WHO STEPPS guidelines. Trained personnel conducted standardized face-to-face interviews to collect sociodemographic, medical history, and lifestyle data, ensuring rigor. CHARLS used multistage stratified probability sampling (150 counties/districts across 28 provinces) to guarantee national representativeness. We set 2015 (Wave 3) as the follow-up baseline (coinciding with the second AIP measurement, essential for CumAIP computation) and 2018 (Wave 4) as the endpoint, forming a 3-year observation window. From 21,095 participants with complete 2012/2015 AIP data, 12,622 were excluded (e.g., missing 2018 outcome/covariates; Fig. 1), leaving 6473 eligible participants for final analysis. These 6473 participants were stratified into four CumAIP quartiles (Q1: lowest 25%, Q4: highest 25%) with sample sizes of 1619, 1618, 1618, and 1618, respectively (Table 2).

Exclusion Criteria:(1) Lost to follow-up during 2012–2018, failing to obtain the 2018 BDRM score (primary outcome);(2) Missing TG and HDL-C data at CHARLS Wave 1 (2012) and Wave 3 (2015), precluding CumAIP calculation (exposure variable);(3) Prior clinical diagnosis of dementia or cognitive impairment. Participants with elevated baseline dementia risk scores (BDRM) were retained to investigate dynamic dementia risk changes, enhancing clinical relevance and generalizability to populations with heterogeneous baseline risk.

### Calculation of CumAIP

AIP was calculated from the logarithm of the ratio of TG to HDL-C obtained from blood samples collected in waves 1 and 3. CumAIP—a proxy for pre-baseline time-weighted cumulative average exposure to dyslipidemia—was calculated as the weighted sum of the average AIP values for each time interval, which was then standardized to the total exposure time (2012–2015).

**Calculation formula:**

① AIP = log10 [TG (mg/dL)/HDL-C (mg/dL)].

② CumAIP = (AIP2012 + AIP2015)/2 * time (2012–2015).

### Determination and definition of middle-aged and elderly dementia and its development

CHARLS lacked direct dementia-related variables (e.g., clinical diagnosis, cognitive tests); thus, this study used the validated BDRM to estimate dementia risk. The BDRM—a population-based study on dementia [15] (Table 1) and performs well across income settings [15, 16], making it suitable for cohorts with limited dementia data. [15]

**Table 1:** Detailed algorithms of BDRM*

BDRM	Definition	Weights
1. Age	Age	
2. History of stroke	We assigned a point of 1 to participants diagnosed with stroke; otherwise, we assigned a point of 0	1.82
3. Subjective memory decline	Memory was estimated by the following questions: “How would you rate your memory at present?” (1) Excellent, (2) Very good, (3) Good, (4) Fair, (5) Poor. Then, we assigned a point of 1 to participants who answered “Poor”; otherwise, we assigned a point of 0	1.31
4. Need for assistance with finances or medications	Needing for assistance with finances or medication was estimated by following questions: “Do you have any difficulties with taking medications?” and “Do you have any difficulties with managing your money?” (1) No, I don’t have any difficulty, (2) I have difficulty but can still do it, (3) Yes, I have difficulty and need help, (4) I cannot do it. Then, we assigned a point of 1 to participants who chose (3) or (4); otherwise, we assigned a point of 0	1.46

*BDRM model yielded a *C* statistic of 0.78 (95% CI = 0.75, 0.81). It performed well in both high-income countries (HICs) and middle-income countries (LMICs)

*BDRM* Rotterdam Study Basic Dementia Risk Model

Developed via Cox proportional hazards modeling, the BDRM includes four weighted risk factors: age (1.09), stroke history (1.82), subjective memory loss (1.31), and need for financial/medication assistance (1.46). Risk scores were calculated by dichotomizing each factor, multiplying by its weight, and summing the values. These factors reflect dementia risk from pathological (age), structural (stroke), symptomatic (memory loss), and functional (assistance need) dimensions; higher scores indicate elevated dementia risk [15].

### Control variables

Covariates in the baseline survey included:(1) Sociodemographic Variables: Including gender (1 = male, 2 = female), age, residential area (1 = rural, 2 = urban), marital status (1 = married cohabiting, 2 = separated, 3 = single/divorced/widowed), education level (1 = elementary school or below, 2 = middle school or above), and annual household expenditure. (2) Lifestyle Variables: Including smoking status (0 = nonsmoker, 1 = smoker) and drinking status (0 = nondrinker, 1 = drinker). (3) Clinical and Metabolic Variables: Including BMI (continuous variable, calculated as weight/height^2^) and number of chronic diseases (count variable, summarized from self-reported noncommunicable diseases: hypertension, dyslipidemia, diabetes, cancer, lung disease, heart problem, kidney disease, asthma, psychological problem, stomach digestive disease, arthritis, liver disease).

### Statistical analysis

Statistical analyses were conducted in R 4.5.0 (two-tailed tests, *α* = 0.05) to ensure methodological rigor for exploring the CumAIP–dementia risk association.

#### Descriptive statistics

(1) Continuous variables: Normally distributed indices (age, BMI, 2015 cognitive function scores, 2018 BDRM scores) were reported as mean ± SD; skewed variables as median (IQR). (2) Categorical variables, including sociodemographic factors, lifestyle factors, and prevalence of self-reported chronic diseases, were described as frequencies (%). (3) Intergroup comparisons: Across CumAIP quartiles, one-way ANOVA (normal continuous), Kruskal–Wallis test (nonparametric), and chi-square test (categorical) were used to identify baseline confounders.

#### Linear regression for CumAIP–dementia risk association

Three hierarchically adjusted linear regression models quantified the association between CumAIP (exposure: time-weighted average of 2012/2015 AIP) and 2018 BDRM scores (outcome):

Model 1:Unadjusted (CumAIP + intercept) for bivariate association;

Model 2:Adjusted for age, sex, residence, education, marital status, and annual household expenditure.

Model 3:Further adjusted for chronic disease count and BMI.

Stroke history was excluded (BDRM component) to avoid double-counting. All models reported *β*-coefficients with 95% CI and robust standard errors (for heteroskedasticity).

#### Association characterization

(1) Nonlinearity test: Restricted cubic splines (3 knots at 10th/50th/90th CumAIP percentiles) in the fully adjusted model; likelihood ratio tests confirmed linearity;(2) Dose–response analysis: CumAIP quartiles (Q1 as reference) analyzed via the three models, with linear trend tests;(3) Stratified analyses: Effect modification evaluated by sex, age (< 60/≥ 60 years), residence, smoking/drinking status, BMI (< 24/≥ 24 kg/m^2^); interaction effects tested via likelihood ratio tests.

#### Validation, missing data and sensitivity analyses

(1) BDRM validation: Spearman’s rank correlation (2015 cognitive function scores vs. 2018 BDRM scores; *r* = − 0.26, *P* < 0.001) to confirm BDRM reliability;(2) Missing data: Handled via multiple imputation (MICE, 20 iterations, < 2% missing) to preserve sample size.

(3) Sensitivity analyses: To confirm result robustness, the following were conducted: (1) quartile-specific complete-case assessments with repeated model fitting to validate dose–response stability; (2) validation against WHO diagnostic criteria [17]to verify outcome reliability; (3) linear regression of CumAIP and 2015 cognitive function scores to explore cognitive function as an intermediate variable; (4) substitution of CumAIP with LDL-C in regression models to compare predictive value.

## Results

### Baseline characteristics of the study population

A total of 6473 participants with complete 2015 baseline data were stratified by CumAIP quartiles (Q1-Q4; *n* = 1619/1618/1618/1618;Table 2). The cohort had a mean age of 61.4 ± 8.5 years, with age decreasing across quartiles (Q1: 62.1 ± 8.8 vs. Q4: 60.7 ± 8.2 years; *P* < 0.001). Males (Q1: 50.3% vs. Q2–Q4: 41.1%–44.1%; *P* < 0.001), rural residents (Q1: 73.0% vs. Q4: 59.3%; *P* < 0.001), and those with elementary/below education (Q1: 72.0% vs. Q4: 66.4%; *P* = 0.001) were more frequent in lower quartiles; married/cohabiting participants were most common in Q4 (86.1%; *P* = 0.012), with no expenditure trend (*P* = 0.156). Current smokers (Q1: 46.2% vs. Q3: 39.4%; *P* = 0.001) and drinkers (Q1: 40.9% vs. Q4: 30.0%; *P* < 0.001) were more prevalent in lower quartiles, while BMI increased (Q1: 22.7 ± 11.9 vs. Q4: 26.2 ± 17.2 kg/m^2^; *P* < 0.001). Prevalence of hypertension (Q1:26.3% vs. Q4:48.0%), diabetes (Q1:5.6% vs. Q4:16.5%), dyslipidemia (Q1:10.6% vs. Q4:33.1%), and heart problems (Q1:14.6% vs. Q4:23.2%) rose with CumAIP (all *P* < 0.001). The 2018 BDRM score decreased across quartiles (Q1:71.7 ± 9.7 vs. Q4:70.3 ± 9.2; *P* < 0.001). These differences were adjusted for in regression models to control confounding.

**Table 2:** Baseline Characteristics of Participants (2015) Stratified by CumAIP Quartiles and Corresponding 2018 Dementia Risk Scores

	Level	Overall	Q1	Q2	Q3	Q4	p
n		6473	1619	1618	1618	1618	
Age (mean (SD))		61.4 (8.5)	62.1 (8.8)	61.7 (8.6)	61.2 (8.4)	60.7 (8.2)	< 0.001
Sex (%)	1	2870 (44.3)	814 (50.3)	713 (44.1)	665 (41.1)	678 (41.9)	< 0.001
	2	3603 (55.7)	805 (49.7)	905 (55.9)	953 (58.9)	940 (58.1)	
Residence (%)	1	4295 (66.4)	1182 (73.0)	1104 (68.2)	1050 (64.9)	959 (59.3)	< 0.001
	2	2178 (33.6)	437 (27.0)	514 (31.8)	568 (35.1)	659 (40.7)	
Marital status (%)	1	5397 (83.4)	1337 (82.6)	1327 (82.1)	1341 (82.9)	1392 (86.1)	0.012
	2	240 (3.7)	64 (4.0)	53 (3.3)	67 (4.1)	56 (3.5)	
	3	833 (12.9)	218 (13.5)	237 (14.7)	210 (13.0)	168 (10.4)	
Education Status (%)	1	4475 (69.1)	1166 (72.0)	1142 (70.6)	1093 (67.6)	1074 (66.4)	0.001
	2	1998 (30.9)	453 (28.0)	476 (29.4)	525 (32.4)	544 (33.6)	
Smoking Status (%)	0	3740 (57.8)	871 (53.8)	950 (58.7)	980 (60.6)	939 (58.0)	0.001
	1	2732 (42.2)	747 (46.2)	668 (41.3)	638 (39.4)	679 (42.0)	
Drinking Status (%)	0	4280 (66.3)	952 (59.1)	1090 (67.5)	1109 (68.7)	1129 (70.0)	< 0.001
	1	2175 (33.7)	659 (40.9)	525 (32.5)	506 (31.3)	485 (30.0)	
Annual Household Expenditure (mean (SD))		12,906.5 (19,384.0)	12,655.6 (20,220.8)	12,319.5 (18,217.6)	12,620.7 (17,906.1)	14,017.6 (21,000.4)	0.156
HTN (%)	0	4039 (63.8)	1173 (73.7)	1080 (68.2)	963 (61.0)	823 (52.0)	< 0.001
	1	2296 (36.2)	418 (26.3)	503 (31.8)	616 (39.0)	759 (48.0)	
Diabetes (%)	0	5647 (89.6)	1490 (94.4)	1460 (91.8)	1386 (88.6)	1311 (83.5)	< 0.001
	1	657 (10.4)	88 (5.6)	130 (8.2)	179 (11.4)	260 (16.5)	
Cancer (%)	0	6277 (98.5)	1573 (99.0)	1570 (98.6)	1563 (98.2)	1571 (98.2)	0.22
	1	96 (1.5)	16 (1.0)	23 (1.4)	28 (1.8)	29 (1.8)	
Lung disease (%)	0	5462 (85.6)	1339 (84.2)	1352 (84.4)	1368 (85.7)	1403 (88.0)	0.009
	1	922 (14.4)	251 (15.8)	250 (15.6)	229 (14.3)	192 (12.0)	
Heart problem (%)	0	5121 (80.8)	1354 (85.4)	1297 (82.2)	1250 (78.9)	1220 (76.8)	< 0.001
	1	1215 (19.2)	231 (14.6)	280 (17.8)	335 (21.1)	369 (23.2)	
Psych problem (%)	0	6252 (97.9)	1562 (97.6)	1562 (97.9)	1556 (97.7)	1572 (98.4)	0.326
	1	135 (2.1)	39 (2.4)	34 (2.1)	37 (2.3)	25 (1.6)	
Arthritis (%)	0	3364 (52.8)	833 (52.2)	816 (51.4)	822 (51.6)	893 (56.0)	0.028
	1	3007 (47.2)	762 (47.8)	773 (48.6)	771 (48.4)	701 (44.0)	
Dyslipidemia (%)	0	4883 (78.8)	1398 (89.4)	1287 (83.7)	1166 (74.9)	1032 (66.9)	< 0.001
	1	1317 (21.2)	165 (10.6)	251 (16.3)	390 (25.1)	511 (33.1)	
Liver disease (%)	0	5889 (92.8)	1460 (92.4)	1474 (93.1)	1471 (92.7)	1484 (93.0)	0.875
	1	457 (7.2)	120 (7.6)	110 (6.9)	116 (7.3)	111 (7.0)	
Kidney disease (%)	0	5699 (89.7)	1420 (89.6)	1423 (89.7)	1408 (88.8)	1448 (90.6)	0.432
	1	656 (10.3)	165 (10.4)	164 (10.3)	177 (11.2)	150 (9.4)	
Stomach digestive disease (%)	0	4245 (66.6)	1036 (65.0)	1033 (64.8)	1075 (67.3)	1101 (69.0)	0.036
	1	2133 (33.4)	557 (35.0)	560 (35.2)	522 (32.7)	494 (31.0)	
Asthma (%)	0	6008 (93.9)	1508 (94.4)	1481 (92.5)	1499 (93.6)	1520 (95.0)	0.02
	1	391 (6.1)	89 (5.6)	120 (7.5)	102 (6.4)	80 (5.0)	
BMI (mean (SD))		24.6 (17.1)	22.7 (11.9)	24.8 (25.7)	24.9 (8.6)	26.2 (17.2)	< 0.001
Dementia (mean (SD))		71.1 (9.5)	71.7 (9.7)	71.4 (9.6)	70.8 (9.4)	70.3 (9.2)	< 0.001

This table presents 2015 baseline characteristics of 6473 participants from CHARLS, stratified by CumAIP quartiles (Q1: lowest 25%, Q4: highest 25%), and includes their 2018 BDRM scores. It compares demographic, lifestyle, clinical, and dementia risk-related variables across CumAIP levels to identify potential confounders for analyzing the CumAIP–BDRM association

Data Presentation: Normally distributed continuous variables (e.g., age, CumAIP, BMI) are expressed as mean (standard deviation); categorical variables (e.g., residence, marital status, smoking status) as n (%).When a variable has sub—categories (e.g., sex, residence), data for each subcategory is presented in separate rows with a “level” indicator

*CumAIP* Cumulative Atherogenic Index of Plasma; *BMI* Body Mass Index; *HTN* Hypertension; *Psych problem* Psychological problem; *Dementia* Rotterdam Basic Dementia Risk Model (BDRM)-derived dementia risk score assessed in 2018

### Relationship between CumAIP and dementia risk score in middle-aged and elderly adults

Linear regression analyses (Table 3) were conducted with the BDRM score as the dependent variable, identifying model-dependent associations between the CumAIP and dementia risk in the study cohort (mean age 61.4 ± 8.5 years). Model 1 (unadjusted) demonstrated a significant negative correlation between CumAIP and BDRM score (*β* = − 0.727, 95% CI − 1.002– − 0.451, *P* < 0.001). After adjusting for sociodemographic confounders (age, sex, residence, education, marital status, annual household expenditure) in Model 2, the association reversed to a significant positive direction (*β* = 0.086, 95% CI 0.049–0.123, *P* < 0.001). Further adjustment for chronic disease count and BMI in Model 3 (fully adjusted) retained this significant positive association (*β* = 0.058, 95% CI 0.018–0.098, *P* = 0.004). Stroke history was excluded from all models to avoid double-counting, as it is a weighted component of the BDRM (Table 1).

**Table 3:** Linear regression analysis showed that the change of CumAIP was associated with the risk of dementia in middle-aged and elderly people

Model	Term	*β*	std.error	Statistic	p.value	conf.low	conf.high
Model1	(Intercept)	71.883	0.198	362.132	0.000	71.494	72.272
Model1	cumAIP	− 0.727	0.141	− 5.167	0.000	− 1.002	− 0.451
Model2	(Intercept)	3.750	0.167	22.459	0.000	3.423	4.078
Model2	cumAIP	0.086	0.019	4.511	0.000	0.049	0.123
Model2	Age	1.098	0.002	538.628	0.000	1.094	1.102
Model2	Sex	0.276	0.033	8.286	0.000	0.211	0.342
Model2	Residence	− 0.194	0.034	− 5.655	0.000	− 0.261	− 0.127
Model2	Education Status	− 0.296	0.037	− 8.067	0.000	− 0.368	− 0.224
Model2	Marital status	− 0.036	0.026	− 1.388	0.165	− 0.088	0.015
Model2	Annual Household Expenditure	− 0.000	0.000	− 2.679	0.007	− 0.000	− 0.000
Model3	(Intercept)	3.937	0.177	22.192	0.000	3.589	4.285
Model3	cumAIP	0.058	0.020	2.848	0.004	0.018	0.098
Model3	Age	1.093	0.002	506.537	0.000	1.089	1.098
Model3	Sex	0.218	0.035	6.216	0.000	0.149	0.286
Model3	Residence	− 0.191	0.0356	− 5.353	0.000	− 0.260	− 0.121
Model3	Education Status	− 0.298	0.038	− 7.839	0.000	− 0.373	− 0.223
Model3	Marital status	− 0.033	0.027	− 1.227	0.220	− 0.087	0.020
Model3	Annual Household Expenditure	− 0.000	0.000	− 2.489	0.013	− 0.000	− 0.000
Model3	HTN	0.106	0.0370	2.855	0.004	0.033	0.178
Model3	Diabetes	0.105	0.057	1.853	0.064	− 0.006	0.216
Model3	Cancer	− 0.218	0.139	− 1.562	0.118	− 0.491	0.056
Model3	Lung disease	− 0.040	0.053	− 0.763	0.445	− 0.145	0.064
Model3	Heart problem	0.194	0.045	4.315	0.000	0.106	0.282
Model3	Psych problem	0.398	0.118	3.378	0.000	0.167	0.629
Model3	Arthritis	0.163	0.034	4.798	0.000	0.097	0.230
Model3	Dyslipidemia	0.172	0.044	3.941	0.000	0.086	0.257
Model3	Liver disease	− 0.002	0.065	− 0.033	0.974	− 0.131	0.126
Model3	Kidney disease	0.145	0.056	2.591	0.010	0.035	0.254
Model3	Stomach digestive disease	0.063	0.036	1.728	0.084	− 0.008	0.134
Model3	Asthma	0.170	0.078	2.186	0.029	0.018	0.323
Model3	BMI	− 0.001	0.000	− 1.523	0.128	− 0.003	0.000

Model Specifications:

Model 1 (Crude Model): Unadjusted (only CumAIP + intercept)

Model 2 (Sociodemographic Adjustment): Adjusted for age, sex, residence, education status, marital status, and annual household expenditure

Model 3 (Fully Adjusted Model): Further adjusted for self-reported chronic diseases (HTN, diabetes, cancer, etc.) and BMI.Note: Stroke history was excluded from adjustment as it is a component of the BDRM dementia risk score

*CumAIP* plasma cumulative atherosclerosis index, *BMI* body mass index, *std.error* Standard error of coefficients, *statistic* test statistic, *conf.low* Lower bound of confidence interval, *cinf.high* Upper bound of confidence interval, *HTN* Hypertension;

Quantitatively, the fully adjusted *β*coefficient indicates that each 1-unit increase in CumAIP (reflecting a 1-unit elevation in long-term atherogenic burden) is associated with a 0.068-unit increase in the BDRM score, even after accounting for sociodemographic factors, lifestyle, and chronic disease burden. The directional shift from a negative (unadjusted) to positive (adjusted) association confirms omitted variable bias in the crude model, supporting CumAIP as an independent factor associated with higher BDRM-estimated dementia risk.

### Subgroup analyses of CumAIP–dementia risk associations

**Fig. 2:**
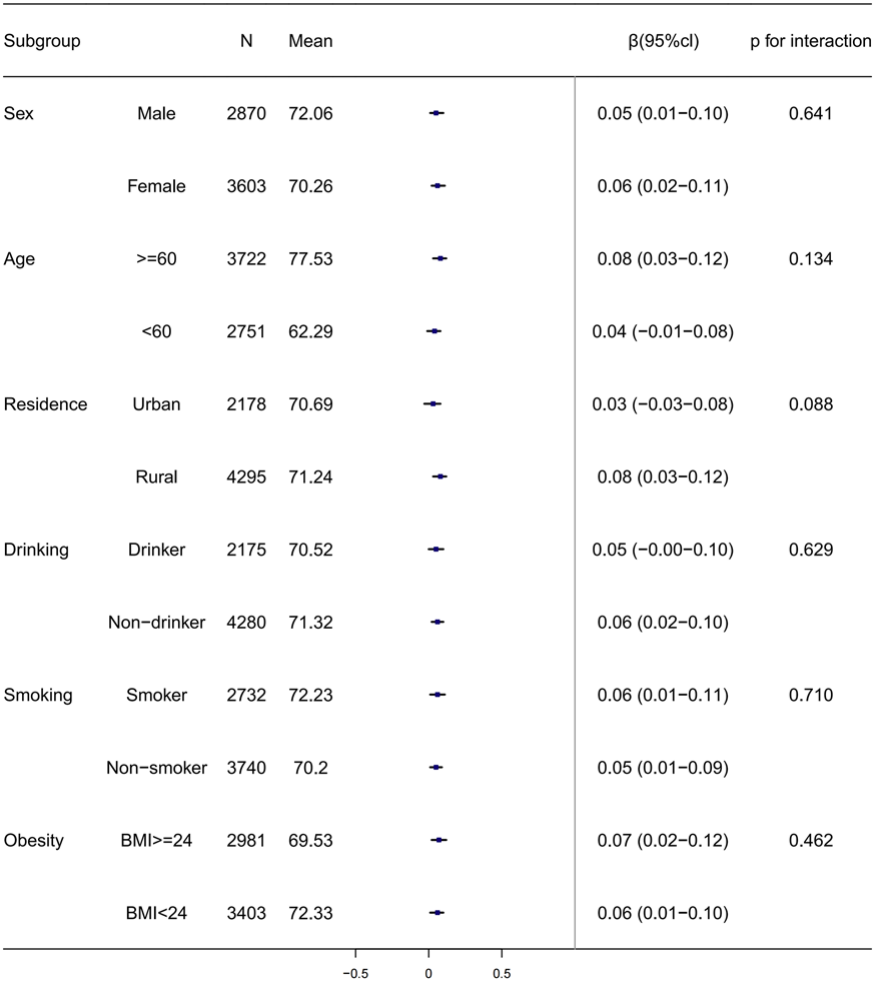
Subgroup analysis of the correlation between CumAIP and BDRM score after 3 years of follow-up. Mean: The average BDRM score. 95% CI 95% confidence interval, if the CI does not include 0, the effect is significant

Subgroup analyses (Table 4), combined with the forest plot (Fig. 2), clearly reveal population heterogeneity in the CumAIP–BDRM association. In the gender stratification, females show a slightly stronger association (*β* = 0.06, 95%CI 0.02–0.11, *P* = 0.006) than males (*β* = 0.05, 95%CI 0.01–0.10, *P* = 0.029), with the former having a longer effect size bar and nonzero-crossing CI in the forest plot. In the age stratification, the strong association in participants aged ≥ 60 years (*β* = 0.08, 95%CI 0.03–0.12, *P* = 0.001) and weak association in those < 60 years (*β* = 0.04, 95%CI − 0.01–0.08, *P* = 0.109) align with the forest plot-only the latter’s CI crosses the null line. Geographically, rural residents exhibit a significant association (*β* = 0.08, 95%CI 0.03–0.12, *P* < 0.001) while urban residents do not (*β* = 0.03, 95%CI − 0.03–0.08, *P* = 0.315), reflected by rural residents’ longer effect size bar and nonzero-crossing CI in Fig. 2.

**Table 4:** Subgroup and interaction analysis of the effect of CumAIP on dementia risk in middle-aged and older adults

	Subgroup	N	Mean_Score	*β*	p_value	P for interaction	conf.low	conf.high
Sex	Male	2870	72.06	0.05	0.029	0.641	0.01	0.10
	Female	3603	70.26	0.06	0.006		0.02	0.11
Age	≥ 60	3722	77.53	0.08	0.001	0.134	0.03	0.12
	< 60	2751	62.29	0.04	0.109		− 0.01	0.08
Residence	Urban	2178	70.69	0.03	0.315	0.088	− 0.03	0.08
	Rural	4295	71.24	0.08	< 0.001		0.03	0.12
Drinking	Drinker	2175	70.52	0.05	0.062	0.629	− 0.00	0.10
	Nondrinker	4280	71.32	0.06	0.005		0.02	0.10
Smoking	Smoker	2732	72.23	0.06	0.011	0.710	0.01	0.11
	Nonsmoker	3740	70.2	0.05	0.023		0.01	0.09
Obesity	BMI ≥ 24	2981	69.53	0.07	0.003	0.462	0.02	0.12
	BMI < 24	3403	72.33	0.06	0.024		0.01	0.10

Definition of acronyms: conf.low/conf.high: 95% confidence interval; mean score: mean score of BDRM dementia risk; BMI: Body Mass Index; *β*:regression coefficient

Among behavioral factors, nondrinkers show a significant association (*β* = 0.06, 95%CI 0.02–0.10, *P* = 0.005) while drinkers have a marginal association (*β* = 0.05, 95%CI − 0.001–0.101, *P* = 0.062)—the forest plot shows nondrinkers’ CI does not cross the null line, while drinkers’ CI is near the null. Both smokers (*β* = 0.06, 95%CI 0.01–0.11, *P* = 0.011) and nonsmokers (*β* = 0.05, 95%CI 0.01–0.09, *P* = 0.023) have significant associations, with smokers’ effect size bar slightly longer in Fig. 2. Metabolically, participants with BMI ≥ 24 kg/m^2^ show a slightly stronger association (*β* = 0.07, 95%CI 0.02–0.12, *P* = 0.003) than those with BMI < 24 kg/m^2^ (*β* = 0.06, 95%CI 0.01–0.10, *P* = 0.024), consistent with the former’s longer effect size bar in the forest plot.

Overall, the forest plot (Fig. 2) intuitively reinforces the effect strength hierarchy from Table 4 via effect size bar length and CI distribution. Together, Table 4 and Fig. 2 confirm that associations are significant (*P* < 0.05) in all subgroups except those < 60 years, urban residents, and drinkers—consistent with nonsignificant interaction effects across all stratifications (Table 4).

### Sensitivity analysis

To validate the robustness of the association between the CumAIP and BDRM-estimated dementia risk, sensitivity analyses were conducted to address potential biases that could affect the primary findings, with the results as follows:

#### Missing data handling

Missing covariates (< 2% of data) were imputed via MICE (20 iterations). Fully adjusted Model 3 showed stable CumAIP coefficient = 0.068 (*P* < 0.001), consistent with primary analysis, ruling out imputation bias (Table 5).

**Table 5:** Sensitivity analysis examines the association between CumAIP stratification and the risk of occurrence

Model	term	*β*	std.error	statistic	p.value	conf.low	conf.high
Model1	(Intercept)	71.883	0.198	362.132	0.000	71.494	72.272
	cumAIP	− 0.726	0.141	− 5.167	0.000	− 1.002	− 0.451
Model2	(Intercept)	3.750	0.167	22.459	0.000	3.423	4.078
	cumAIP	0.086	0.019	4.511	0.000	0.049	0.123
	Age	1.099	0.002	538.628	0.000	1.094	1.102
	Sex	0.276	0.033	8.285	0.000	0.211	0.342
	Residence	− 0.194	0.034	− 5.655	0.000	− 0.261	− 0.127
	Education status	− 0.296	0.037	− 8.067	0.000	− 0.368	− 0.224
	Marital status	− 0.036	0.026	− 1.389	0.165	− 0.088	0.015
	Annual household expenditure	− 0.000	0.000	− 2.679	0.007	− 0.000	− 0.000
Model3	(Intercept)	3.656	0.168	21.803	0.000	3.327	3.985
	cumAIP	0.068	0.019	3.552	0.000	0.030	0.105
	Age	1.096	0.002	537.323	0.000	1.092	1.100
	Sex	0.274	0.033	8.244	0.000	0.209	0.339
	Residence	− 0.184	0.034	− 5.400	0.000	− 0.251	− 0.117
	Education status	− 0.288	0.036	− 7.902	0.000	− 0.359	− 0.217
	Marital status	− 0.031	0.026	− 1.170	0.242	− 0.082	0.021
	Annual household expenditure	− 0.000	0.000	− 2.834	0.004	− 0.000	− 0.000
	Number of chronic conditions	0.189	0.021	9.048	0.000	0.148	0.230
	BMI	− 0.000	0.001	− 1.184	0.236	− 0.003	0.000

Model 1: Coarse model;

Model 2 adjusts for age, gender, place of residence, education level, marital status and annual household expenditure;

Model 3 adjusts for age, gender, place of residence, education level, marital status, annual household expenditure, number of chronic diseases, and BMI;

#### Intermediate pathway and alternative marker validation

**Fig. 3:**
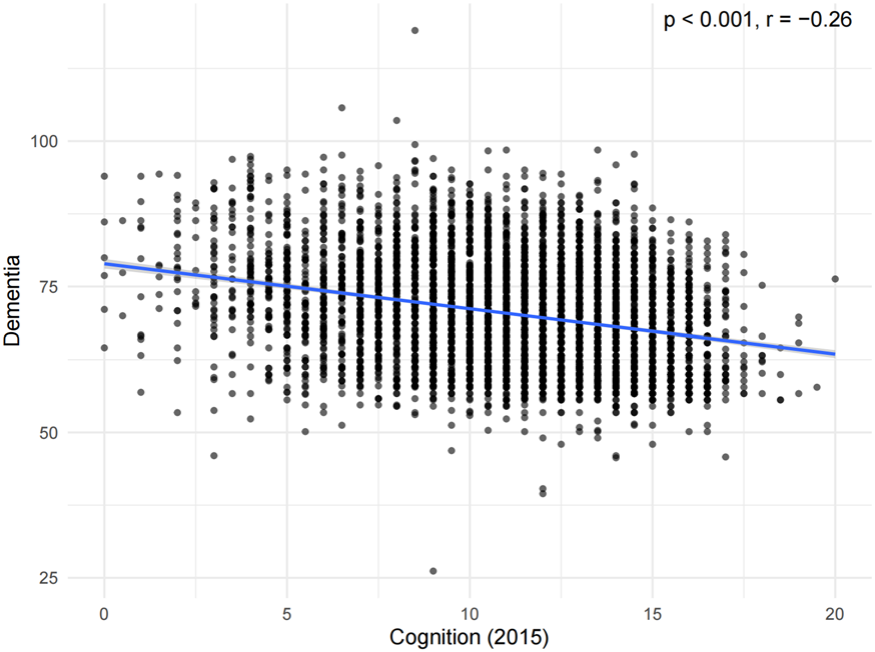
Correlation Analysis between 2015 Cognitive Function Score and Dementia Risk Score
  Linear regression (Table 6):In the fully adjusted model, CumAIP correlated positively with 2015 cognitive function scores (higher scores indicate better cognitive function; *β* = 0.123, 95% CI 0.008–0.237, *P* = 0.037), indicating that higher CumAIP is linked to worse cognitive status.

1. To verify the hypothesized “CumAIP → cognitive function → dementia” pathway, two complementary analyses were conducted:
  Correlation visualization (Fig. 3):Spearman correlation scatter plot demonstrated a significant negative association between 2015 cognitive function scores and BDRM-estimated dementia risk scores (*r* = − 0.26, *P* < 0.001), confirming that poorer cognitive function correlates with higher dementia risk and laying the foundation for cognitive function as a plausible intermediate link.

**Table 6:** Sensitivity analysis: linear regression of CumAIP and 2015 Cognitive Function Score in CHARLS participants

Model	Term	*β*	std.error	Statistic	p.value	conf.low	conf.high
Model1	(Intercept)	10.687	0.076	140.101	0.000	10.538	10.837
Model1	cumAIP	0.292	0.054	5.404	0.000	0.186	0.398
Model2	(Intercept)	11.873	0.482	24.622	0.000	10.928	12.819
Model2	cumAIP	0.125	0.055	2.250	0.025	0.016	0.233
Model2	Age	− 0.065	0.006	− 10.898	0.000	− 0.077	− 0.053
Model2	Sex	− 0.759	0.096	− 7.940	0.000	− 0.947	− 0.572
Model2	Residence	0.926	0.097	9.512	0.000	0.735	1.117
Model2	Education Status	2.309	0.102	22.745	0.000	2.110	2.509
Model2	Marital status	− 0.226	0.077	− 2.920	0.004	− 0.377	− 0.074
Model2	Annual Household Expenditure	0.000	0.000	0.789	0.430	− 0.000	0.000
Model3	(Intercept)	11.437	0.508	22.533	0.000	10.442	12.432
Model3	cumAIP	0.123	0.059	2.092	0.037	0.008	0.237
Model3	Age	− 0.061	0.006	− 9.676	0.000	− 0.073	− 0.048
Model3	Sex	− 0.759	0.099	− 7.651	0.000	− 0.954	− 0.565
Model3	Residence	0.885	0.100	8.813	0.000	0.688	1.082
Model3	Education status	2.364	0.105	22.568	0.000	2.159	2.570
Model3	Marital status	− 0.233	0.080	− 2.909	0.004	− 0.389	− 0.076
Model3	Annual household expenditure	0.000	0.000	0.820	0.413	− 0.000	0.000
Model3	HTN	0.040	0.103	0.384	0.701	− 0.163	0.242
Model3	Diabetes	− 0.145	0.157	− 0.927	0.354	− 0.453	0.162
Model3	Cancer	0.060	0.393	0.151	0.880	− 0.712	0.831
Model3	Stomach digestive disease	0.057	0.101	0.570	0.569	− 0.140	0.255
Model3	Asthma	− 0.366	0.198	− 1.850	0.064	− 0.754	0.022
Model3	BMI	0.007	0.003	2.871	0.004	0.002	0.012

Model Design:

Model 1 (Crude): Unadjusted (CumAIP + intercept)

Model 2 (Sociodemographic Adjustment): Adjusted for age, sex, residence, education, marital status, annual household expenditure

Model 3 (Fully Adjusted): Further adjusted for chronic diseases (HTN, diabetes, etc.) and BMI

*CumAIP* Cumulative Atherogenic Index of Plasma; *BDRM* Rotterdam Basic Dementia Risk Model; *CHARLS* China Health and Retirement Longitudinal Study; *HTN* Hypertension; *BMI* Body Mass Index; *β* Regression coefficient; *CI* Confidence Interval
2. To highlight CumAIP’s incremental value over traditional markers, substituting CumAIP with LDL-C revealed a weaker BDRM association (*β* = 0.019, *P* < 0.001 vs. CumAIP *β* = 0.068), confirming CumAIP captures more comprehensive atherogenic burden (Table 7).

**Table 7:** Sensitivity analysis: linear regression of LDL-C and BDRM score in CHARLS Chinese adults (mean age 61.4 ± 8.5 years)

Model	Term	*Β*	std.error	Statistic	p.value	conf.low	conf.high
Model1	(Intercept)	69.508	0.432	160.757	0.000	68.660	70.356
Model1	ldl	0.015	0.004	3.718	0.000	0.007	0.023
Model2	(Intercept)	69.450	0.631	110.004	0.000	68.212	70.688
Model2	ldl	0.020	0.005	4.291	0.000	0.011	0.030
Model2	Residence	− 0.589	0.289	− 2.036	0.042	− 1.156	− 0.022
Model2	Annual Household Expenditure	− 0.000	0.000	− 3.932	0.000	− 0.000	− 0.000
Model3	(Intercept)	68.226	0.703	97.025	0.000	66.847	69.604
Model3	ldl	0.019	0.005	3.800	0.000	0.009	0.028
Model3	Residence	− 0.634	0.301	− 2.105	0.035	− 1.224	− 0.043
Model3	Annual Household Expenditure	− 0.000	0.000	− 3.048	0.002	− 0.000	− 0.000
Model3	HTN	2.674	0.315	8.481	0.000	2.056	3.292
Model3	Diabetes	1.144	0.488	2.342	0.019	0.186	2.102
Model3	Cancer	− 0.022	1.208	− 0.018	0.985	− 2.390	2.346
Model3	Lung disease	0.908	0.461	1.971	0.049	0.005	1.812
Model3	Heart problem	1.959	0.387	5.068	0.000	1.201	2.717
Model3	Psych problem	0.780	1.020	0.765	0.444	− 1.220	2.781
Model3	Arthritis	0.900	0.293	3.073	0.002	0.326	1.474
Model3	Dyslipidemia	− 0.714	0.372	− 1.917	0.055	− 1.443	0.016
Model3	Liver disease	− 1.039	0.567	− 1.835	0.067	− 2.150	0.071
Model3	Kidney disease	0.268	0.483	0.555	0.579	− 0.678	1.214
Model3	Stomach digestive disease	− 0.730	0.314	− 2.327	0.020	− 1.345	− 0.115
Model3	Asthma	2.473	0.674	3.668	0.000	1.151	3.795
Model3	BMI	− 0.015	0.007	− 2.198	0.028	− 0.028	− 0.002

This table presents sensitivity analysis results for 6473 CHARLS participants, using LDL-C (continuous exposure, mg/dL) (instead of primary CumAIP) to validate lipid–dementia risk association robustness. Outcome: 2018 BDRM score

Aim: Confirm consistency between LDL-C (traditional atherogenic marker) and CumAIP findings, and highlight CumAIP’s incremental value. Three models mirror the primary framework; stroke history excluded (weighted BDRM component) to avoid double-counting bias

*LDL-C* Low-Density Lipoprotein Cholesterol; *CumAIP* Cumulative Atherogenic Index of Plasma; *BDRM* Rotterdam Study Basic Dementia Risk Model; *CHARLS* China Health and Retirement Longitudinal Study; *β* Regression coefficient; *CI* Confidence Interval; *HTN* Hypertension; *BMI* Body Mass Index

Figure 3 is a scatter plot showing the Spearman rank correlation between participants’ cognitive function scores and dementia risk scores. The x-axis represents 2015 cognitive performance, with higher scores indicating better cognitive function. The y-axis denotes dementia risk scores, derived from the BDRM, where higher scores mean increased future dementia risk.

Spearman analysis revealed a significant inverse association: Spearman’s *r* = − 0.26, *P* < 0.001. Specifically, higher 2015 cognitive scores correlated with lower dementia risk scores, and vice versa. *P* < 0.001 confirms the robustness of this moderate negative correlation.

This finding supports the study’s core logic: as CumAIP is hypothesized to contribute to dementia via cognitive impairment-related pathways, the observed correlation validates cognitive function as a plausible intermediate link between CumAIP and dementia risk, and reinforces cognitive assessments for dementia risk stratification in the target population.

### Linear association between CumAIP and dementia scores and verification of dose–response relationship

#### Nonlinear test (verifying linear relationship, Table 8)

**Table 8:** Nonlinearity test

Factor	Chi-Square	d.f	P
cumAIP	6.34	3	0.0963
Nonlinear	0.07	2	0.9671
Sex	74.6	1	< 0.0001
Marital status	331.95	1	< 0.0001
Education Status	614.03	1	< 0.0001
Smoking Status	12.74	1	0.0004
Drinking Status	43.57	1	< 0.0001
TOTAL	1126.25	8	< 0.0001

The P value of the nonlinear term (0.9671) is much greater than 0.05, indicating that there is insufficient evidence to support a nonlinear relationship between CumAIP and dementia. Even though the total effect is close to significance (*P* = 0.0963), the nonlinear component contributes nothing, so the impact of CumAIP on dementia is more consistent with a linear trend

The association pattern between CumAIP and dementia scores was analyzed using RCS curves. Results showed that the specific test for nonlinear terms yielded a *P* value of 0.9671 (far exceeding 0.05), indicating insufficient evidence to support a nonlinear relationship between the two. Although the overall test for deviation from linearity approached marginal significance (*P* = 0.0963), the noncontributory role of nonlinear components, combined with linear regression results (Model 3: *β* = 0.068, 95% CI 0.030–0.105, *P* < 0.001), confirmed a continuous linear association between CumAIP and dementia scores, laying the foundation for subsequent dose–response relationship analyses.

#### Quartile results (analyzing dose–response relationship, Table 9)

**Table 9:** Quartile results (analyzing dose–response relationship)

	Model	Term	*β*	Std.Error	Statistic	P.Value	Conf.Low	Conf.High
1	Model1 (Unadjusted)	Q2 versus Q1	− 0.382	0.333	− 1.147	0.251	− 1.034	0.271
2	Model1 (Unadjusted)	Q3 versus Q1	− 0.932	0.333	− 2.801	0.005	− 1.584	− 0.28
3	Model1 (Unadjusted)	Q4 versus Q1	− 1.448	0.333	− 4.352	< 0.001	− 2.1	− 0.796
4	Model2 (Adjusted for Demographics)	Q2 versus Q1	0.023	0.045	0.521	0.602	− 0.065	0.112
5	Model2 (Adjusted for Demographics)	Q3 versus Q1	0.053	0.045	1.172	0.241	− 0.035	0.141
6	Model2 (Adjusted for Demographics)	Q4 versus Q1	0.154	0.045	3.401	< 0.001	0.065	0.242
7	Model3 (Fully Adjusted)	Q2 versus Q1	− 0.003	0.046	− 0.063	0.949	− 0.094	0.088
8	Model3 (Fully Adjusted)	Q3 versus Q1	0.009	0.047	0.189	0.85	− 0.082	0.1
9	Model3 (Fully Adjusted)	Q4 versus Q1	0.103	0.048	2.159	0.031	0.009	0.197
10	Trend Test (Model1)	Linear Trend (Q1 → Q4)	− 1.094	0.235	− 4.652	< 0.001	− 1.556	− 0.633
11	Trend Test (Model2)	Linear Trend (Q1 → Q4)	0.11	0.032	3.429	< 0.001	0.047	0.172
12	Trend Test (Model3)	Linear Trend (Q1 → Q4)	0.072	0.034	2.117	0.034	0.005	0.139

*CumAIP* Cumulative Atherogenic Index of Plasma; *BDRM* Rotterdam Study Basic Dementia Risk Model; *Q1–Q4* CumAIP quartiles (Q1 = reference); *β* Regression coefficient; *CI* Confidence Interval

To determine the dose–response between CumAIP and BDRM scores, CumAIP was stratified into quartiles (Q1:lowest 25%, reference; Q2-Q4: ascending). Linear regression (3 adjusted models) and trend tests showed:(1) Effect Direction & Confounders: Model1:CumAIP negatively correlated with BDRM (Q4 vs Q1: *β* = − 1.448, 95%CI − 2.100– − 0.796, *P* < 0.001). Model2:association reversed to positive (Q4 vs. Q1: *β* = 0.154, 95%CI 0.065–0.242, *P* < 0.001). Model 3: Q4 vs. Q1 remained significant (*β* = 0.103, 95%CI 0.009–0.197, *P* = 0.031); Q2/Q3 nonsignificant (P > 0.05). Sociodemographics are key confounders;(2) Q4-Specific Threshold Effect: Only Q4 associated with Q1: Model 3 showed Q4’s BDRM score 0.103 units higher than Q1 (*P* = 0.031); Q2 (*β* = − 0.003, *P* = 0.949) and Q3 (*β* = 0.009, *P* = 0.850) had no differences. CumAIP’s risk effect emerges above Q4;(3) Linear Trend: All models had upward trends: Model 1 (trend *β* = − 1.094, *P* < 0.001, confounder-biased), Model 2 (trend *β* = 0.110, 95%CI 0.047–0.172, *P* < 0.001), Model 3 (trend *β* = 0.072, 95%CI 0.005–0.139, *P* = 0.034). Aligns with nonlinearity test (nonlinear term *P* = 0.9671, Table 8), confirming linear dose–response; (4) Clinical Significance: CumAIP better identifies high-exposure risk than single lipid tests. Prioritize lipid monitoring/intervention for Q4 individuals; Q4 serves as a risk stratification threshold.

#### Subgroup analysis (quartile-based, verifying association stability, Table 10)

**Table 10:** Subgroup analysis (quartile-based, verifying association stability)

	Stratification	Subgroup	Term	*β*	P.Value	Conf.Low	Conf.High	Trend_P
1	Sex	Male	cumAIP_q4Q2	0.19	< 0.001	0.08	0.3	0.056
2	Sex	Male	cumAIP_q4Q3	0.08	0.135	− 0.03	0.19	
3	Sex	Male	cumAIP_q4Q4	0.15	0.008	0.04	0.26	
4	Sex	Female	cumAIP_q4Q2	0.01	0.815	− 0.09	0.12	0.001
5	Sex	Female	cumAIP_q4Q3	0.07	0.192	− 0.03	0.17	
6	Sex	Female	cumAIP_q4Q4	0.16	0.003	0.06	0.26	
7	Age	≥ 60	cumAIP_q4Q2	0.06	0.25	− 0.04	0.17	0.002
8	Age	≥ 60	cumAIP_q4Q3	0.09	0.106	− 0.02	0.19	
9	Age	≥ 60	cumAIP_q4Q4	0.17	0.002	0.06	0.28	
10	Age	< 60	cumAIP_q4Q2	0	0.999	− 0.1	0.1	0.143
11	Age	< 60	cumAIP_q4Q3	0.06	0.28	− 0.05	0.16	
12	Age	< 60	cumAIP_q4Q4	0.06	0.235	− 0.04	0.17	
13	Residence	Urban	cumAIP_q4Q2	− 0.01	0.817	− 0.14	0.11	0.762
14	Residence	Urban	cumAIP_q4Q3	− 0.03	0.615	− 0.15	0.09	
15	Residence	Urban	cumAIP_q4Q4	0.03	0.682	− 0.1	0.15	
16	Residence	Rural	cumAIP_q4Q2	0.08	0.078	− 0.01	0.18	< 0.001
17	Residence	Rural	cumAIP_q4Q3	0.1	0.043	0	0.19	
18	Residence	Rural	cumAIP_q4Q4	0.17	< 0.001	0.08	0.27	
19	Drinking	Drinker	cumAIP_q4Q2	0.13	0.025	0.02	0.25	0.049
20	Drinking	Drinker	cumAIP_q4Q3	0.04	0.463	− 0.07	0.16	
21	Drinking	Drinker	cumAIP_q4Q4	0.16	0.01	0.04	0.28	
22	Drinking	Nondrinker	cumAIP_q4Q2	0.07	0.16	− 0.03	0.16	0.003
23	Drinking	Nondrinker	cumAIP_q4Q3	0.08	0.084	− 0.01	0.18	
24	Drinking	Nondrinker	cumAIP_q4Q4	0.15	0.002	0.05	0.25	
25	Smoking	Smoker	cumAIP_q4Q2	0.14	0.013	0.03	0.26	0.043
26	Smoking	Smoker	cumAIP_q4Q3	0.06	0.295	− 0.05	0.17	
27	Smoking	Smoker	cumAIP_q4Q4	0.15	0.009	0.04	0.27	
28	Smoking	Nonsmoker	cumAIP_q4Q2	0.02	0.666	− 0.08	0.12	0.018
29	Smoking	Nonsmoker	cumAIP_q4Q3	0.06	0.248	− 0.04	0.16	
30	Smoking	Nonsmoker	cumAIP_q4Q4	0.12	0.024	0.02	0.22	
31	Obesity	BMI ≥ 24	cumAIP_q4Q2	0.13	0.022	0.02	0.24	0.028
32	Obesity	BMI ≥ 24	cumAIP_q4Q3	0.04	0.483	− 0.07	0.15	
33	Obesity	BMI ≥ 24	cumAIP_q4Q4	0.16	0.005	0.05	0.28	
34	Obesity	BMI < 24	cumAIP_q4Q2	0.11	0.028	0.01	0.21	0.019
35	Obesity	BMI < 24	cumAIP_q4Q3	0.07	0.153	− 0.03	0.18	
36	Obesity	BMI < 24	cumAIP_q4Q4	0.14	0.007	0.04	0.25	

After stratification by sex, age, residence, drinking/smoking status, and obesity, most subgroup results aligned with the overall analysis: significant positive associations between CumAIP Q4 and Q1 were observed in key subgroups, while no significance was found in age < 60 or urban residence; Q2/Q3 showed no significant effects versus Q1 across all subgroups. Linear trend tests were significant (*P* < 0.05) except in age < 60 and urban residence, and no significant interaction effects were detected (all *P*-interaction > 0.05), confirming the CumAIP-BDRM dose–response is stable in most subgroups and unmodified by stratifying factors.

#### Sensitivity analysis (verifying result robustness, Table 11)

**Table 11:** Sensitivity analysis (verifying result robustness)

Model	Term	*β*	Std.Error	Statistic	P.Value	Conf.Low	Conf.High	p for trend
Model1 (Unadjusted)	Q2 versus Q1	− 0.382	0.333	− 1.147	0.251	− 1.034	0.271	< 0.001
Model1 (Unadjusted)	Q3 versus Q1	− 0.932	0.333	− 2.801	0.005	− 1.584	− 0.28	
Model1 (Unadjusted)	Q4 versus Q1	− 1.448	0.333	− 4.352	< 0.001	− 2.1	− 0.796	
Model2 (Adjusted for Demographics)	Q2 versus Q1	0.043	0.038	1.132	0.258	− 0.032	0.119	< 0.001
Model2 (Adjusted for Demographics)	Q3 versus Q1	0.084	0.039	2.185	0.029	0.009	0.16	
Model2 (Adjusted for Demographics)	Q4 versus Q1	0.162	0.039	4.180	< 0.001	0.086	0.238	
Model3 (Fully Adjusted)	Q2 versus Q1	0.034	0.038	0.884	0.377	− 0.041	0.108	0.001
Model3 (Fully Adjusted)	Q3 versus Q1	0.051	0.038	1.337	0.181	− 0.024	0.126	
Model3 (Fully Adjusted)	Q4 versus Q1	0.124	0.039	3.223	0.001	0.049	0.2	

To further validate the robustness of the CumAIP-BDRM linear and dose–response relationships (avoiding biases from analytical methods), sensitivity analyses (complete case analysis, alternative covariate adjustment, modified CumAIP definitions) were conducted. Results remained stable: linear regression *β* values for CumAIP-BDRM association were consistent; Q4 (vs. Q1) significant effect persisted (fully adjusted: *β* = 0.124, *P* = 0.001), and trend tests stayed significant (demographic-adjusted: *P* < 0.001; fully adjusted: *P* = 0.001). This confirms core conclusions are highly robust to analytical variations.

In summary, data from the tables sequentially confirm the conclusion that “CumAIP is linearly associated with BDRM-estimated dementia risk scores and exhibits a dose–response relationship concentrated in the highest quartile.” These results remain stable and robust across most subgroups and all sensitivity analyses.

## Discussion

### Clinical and mechanistic insights into the association between CumAIP and BDRM-estimated dementia risk in the CHARLS cohort

The study identifying a positive association between CumAIP (a proxy for sustained pre-baseline time-weighted cumulative dyslipidemia exposure) and BDRM-estimated dementia risk in Chinese adults ~ 60 years old. The clinically meaningful association—Q4 (highest CumAIP) had higher BDRM scores than Q1, with stability verified by sensitivity analysis—highlights CumAIP’s advantage over single lipid measurements in capturing long-term dyslipidemia exposure for dementia risk assessment. Subgroup analysis showed the association was more pronounced in females, age ≥ 60 years, rural residents, nondrinkers, and overweight/obese populations (nonsignificant in age < 60 years or urban residents); most interaction *P* values > 0.05. The association persisted after adjusting for demographics, lifestyle, and chronic diseases, supporting CumAIP as an independent factor for BDRM-estimated dementia risk, with multi-dimensional sensitivity analyses confirming conclusion robustness.

Consistent with previous studies, Dementia, Alzheimer’s disease, and cardiovascular and cerebrovascular diseases such as stroke are closely linked [18–20]. A cross-sectional study of the AIP index found that middle-aged and elderly people with AIP ≥ 2.32 had a 40% increased risk of developing CMD [21], indicating that high-level AIP exposure is an independent risk factor for CMD. This study further reveals that its cumulative effect is also applicable to dementia risk prediction. However, Kumaradev [22] and others found a weak association between dynamic changes in AIP and cognitive decline in the European and American populations, which may reflect differences in population heterogeneity, follow-up time, different standards used to define dementia, outcome measurements, and confounding factors.

Globally, dementia affects an estimated 47 million individuals at present, with projections indicating this figure will surge to 131 million by 2050 [23]. Notably, cerebrovascular disease stands as the most prevalent comorbidity in Alzheimer’s disease (AD) [24], yet definitive evidence of cerebrovascular involvement is detected in only around 5% of all dementia cases. Therefore, in the diagnosis and treatment of dementia, medical conditions that may affect cognition should be the focus, including risk factors for vascular disease, existing brain conditions, and the use of drugs that can impair cognition (e.g., hypnotics and anxiolytics like benzodiazepines; analgesics such as codeine-containing preparations; anticholinergic drugs such as tricyclic antidepressants and bladder antimuscarinics) [25]. This strategy aids in early identification of cognitive impairment, such as MCI (mild cognitive impairment) accompanied by executive dysfunction.

This study adopted the longitudinal approach by Smith JP and other [26, 27], to calculate the continuous variable CumAIP for analyzing its association with dementia risk in adults ~ 60 years old. Prioritizing dynamic lipid monitoring for subgroups with pronounced CumAIP effects aligns with China’s”Healthy Aging” strategy. Scaling such interventions could reduce projected elderly dementia prevalence by 2050 and alleviate associated socioeconomic burden (exceeding 1% of annual GDP [3]).

Notably, as our study links CumAIP to dementia risk, its potential relevance to AD—the main cause of dementia—helps clarify this association. AD pathogenesis involves *β*-amyloid (A*β*) deposition, hyperphosphorylated Tau neurofibrillary tangles, and inflammation [28, 29], with fibrillar A*β* inducing neuronal dysfunction/death via microglial activation and inflammatory mediator release [30]. Yet current dementia pharmacotherapies only improve symptoms (minimal long-term impact on hospitalization or mortality), highlighting the need for early biomarkers (e.g., CumAIP) and dynamic monitoring to slow dementia progression.

In addition, consistent validation studies have confirmed that AIP serves as a robust indicator of atherosclerosis and cardiovascular disease risk [31–33]. As an indicator of elevated coronary artery disease (CAD) risk independent of smoking history, diabetes, and hypertension, AIP may offer greater predictive value than individual lipid parameters [34]. Dobiásová et al. [8] established a direct correlation between AIP and coronary angiographic features in patients with CAD, thereby verifying a robust link between AIP and atherosclerosis—along with its vascular complications—at the interventional level. This finding was further supported by a Moroccan study, which demonstrated a significant association between AIP and CIMT, a well-recognized surrogate marker for atherosclerosis [35].

Collectively, this study demonstrates that CumAIP (the pre-baseline time-weighted average of 2012–2015 CHARLS AIP values, which reflects long-term sustained dyslipidemia) exhibits an independent positive association with BDRM-estimated dementia risk in Chinese adults participating in the CHARLS cohort, with a mean age of 61.4 ± 8.5 years. Three evidence-based but validation-requiring pathways are proposed to explain this association: CumAIP-related dyslipidemia accelerates atherosclerosis (stronger in rural residents, *β* = 0.08 vs. urban *β* = 0.03, Table 4)—consistent with 42% untreated dyslipidemia in rural areas (CHARLS 2018)—and reduces cerebral blood flow; cumulative atherogenic burden elevates inflammation [36] and neuroinflammation; long-term dyslipidemia induces insulin resistance [37] and disrupts brain glucose metabolism.

Stratified analyses, sensitivity tests, and validation of linearity (nonlinear term *P* = 0.9671, Table 8) and dose–response patterns (Q4 vs. Q1:*β* = 0.103, *P* = 0.031, Table 9) confirm result robustness, with the CumAIP-BDRM association most notable in females, adults ≥ 60 years, and rural residents. CumAIP outperforms LDL-C (*β* = 0.058 vs. 0.019, Table 7) in dementia risk prediction, supporting targeted lipid monitoring in these high-risk subgroups to align with China’s dementia prevention goals amid aging.

## Strengths and limitations

Several key strengths of this study contribute to the reliability and relevance of its findings:(1) Innovative exposure assessment: First to use time-weighted CumAIP for dementia risk evaluation in Asian adults. CumAIP captures sustained dyslipidemia, avoiding regression dilution bias via three-wave data, and remains an independent BDRM predictor (fully adjusted Model 3: *β* = 0.068, *P* < 0.001; Table 5). (2) Biologically plausible associations: Linear (nonlinear term *P* = 0.9671;Table 8) and dose–response (Q4 vs. Q1: *β* = 0.103, *P* = 0.031;Table 9) relationships between CumAIP and BDRM scores support mechanisms like vascular injury and neuroinflammation. (3) Targeted subgroup insights: Stratified analyses identified high-risk groups (females, ≥ 60 years, rural residents; Tables 4 and 10) to guide precision lipid monitoring. (4) Methodological rigor: Missing data (< 2%) handled via MICE (20 iterations) with stable results (*β* = 0.068, *P* < 0.001; Table 5); Progressive adjustment (Model 1 → 3) confirmed CumAIP independence (*β*: − 0.727–0.068, *P* < 0.001; Table 3);Sensitivity analyses (complete-case, LDL-C substitution) validated robustness (LDL-C *β* = 0.019 vs. CumAIP *β* = 0.068; Table 7). (5) Clinical translatability: CumAIP uses routine TG/HDL-C data, with quantifiable targets (e.g., 0.5-unit CumAIP reduction linked to 0.034-unit BDRM decrease) for primary care.

However, this study also has some limitations:(1) Outcome measure limitations: The BDRM relies on demographic and self-reported variables. Thus, the CumAIP–BDRM association reflects estimated, not clinically confirmed dementia (e.g., DSM-5/NIA-AA), which limits generalizability to verified cases. In addition, potential recall bias in self-reported BDRM components may introduce residual confounding. (2) Temporal overlap in CumAIP calculation and outcome follow-up: A limitation is that while our CumAIP uses a validated cumulative assessment method [27, 28], it incorporates 2015 AIP data overlapping with the 2015 baseline of 2018 dementia risk follow-up, requiring further strictly prospective studies (3) Unmeasured genetic and vascular markers: Key variables (apolipoprotein E [ApoE] genotype, brachial-ankle pulse wave velocity [baPWV]) were not measured. ApoE genotype, a major genetic determinant of dementia, was unaccounted for, leaving inherent genetic susceptibility unadjusted. BaPWV absence precluded exploration of arterial stiffness as a mediator between CumAIP and dementia, limiting validation of the underlying vascular mechanism and depth of causal inference;(4) Uncollected drug-related data: Detailed information on participants’ use of drugs affecting cognitive function, blood lipids, or blood glucose (e.g., lipid-lowering medications, anticholinergic drugs) was not proactively collected. This prevented adjustment for potential confounding—for example, lipid-lowering drugs may reduce CumAIP while modifying dementia risk, introducing bias that compromises the objectivity of the CumAIP–dementia association;(5) Unassessed collinearity: Despite confirming CumAIP-BDRM linearity (*P* = 0.9671; Table 8) and excluding stroke history (a BDRM component) from models to avoid bias, potential correlations between CumAIP and other BDRM subdivisions were unevaluated, leaving residual confounding unruled out. Future studies should assess this via VIF;(6) Inadequate smoking/alcohol exposure detail: Smoking and alcohol were only dichotomized without quantifying intensity (e.g., daily cigarettes, weekly alcohol intake). This obscures subgroup differences in the CumAIP–dementia association and introduces residual confounding.

## Conclusions

This prospective CHARLS-based study of Chinese adults (mean age 61.4 ± 8.5 years) confirms that CumAIP—a marker of long-term dyslipidemia—is independently associated with higher BDRM-estimated dementia risk. This association is linear (nonlinear term *P* = 0.9671) and dose-dependent (highest vs. lowest CumAIP quartile showing elevated BDRM scores), with each 1-unit CumAIP increase correlating with higher BDRM scores in the fully adjusted model.

Notably, the association is more pronounced in females, rural residents, nondrinkers, smokers, and those with BMI ≥ 24 kg/m^2^. These findings support CumAIP’s utility for dementia risk stratification and highlight targeted dynamic lipid monitoring in these high-risk groups to reduce BDRM-estimated dementia risk.

## Data Availability

Data will be provided upon request.

## Abbreviations

CHARLS: China Health and Retirement Longitudinal Study
CumAIP: Cumulative Atherogenic Index of Plasma
BMI: Body Mass Index
BDRM: Rotterdam Study Basic Dementia Risk Model
CMD: Cardiometabolic Diseases
CI: Confidence Interval
TG: Triglycerides
TC: Total Cholesterol
LDL-C: Low-Density Lipoprotein Cholesterol
CAD: Coronary artery disease
RCS: Restricted cubic spline
ANOVA: Analysis of Variance
baPWV: Brachial-ankle pulse wave velocity
